# Studying the prevalence of ApoE Haplotypes in the Caribbean Hispanics from Puerto Rico

**DOI:** 10.1002/alz.095426

**Published:** 2025-01-09

**Authors:** Frances M Marin Maldonado, Cristal Garcia Blondet, Ana S Duconge, Jessica Renta, Jose Colon Saez, Jorge Duconge Soler

**Affiliations:** ^1^ School of Pharmacy University of Puerto Rico, San Juan, PR USA; ^2^ University of Puerto Rico at Rio Piedras, San Juan, PR USA; ^3^ School of Medicine University of Puerto Rico, San Juan, PR USA

## Abstract

**Background:**

Among different ethnic groups, Hispanics rank second in Alzheimer’s disease (AD) prevalence. However, most studies on this condition have been conducted in Europeans. To address this bias in representation we interviewed and collected samples from Caribbean Hispanics living in Puerto Rico; to ascertain the prevalence of *ApoE* haplotypes and measure their association with the development of AD in this population.

**Method:**

Initially, 150 Caribbean Hispanic adults participated in this study (females = 83, males = 68), divided into three groups (n = 50 each): cognitively normal (CN) participants, cardiovascular (CV) and Alzheimer’s disease (AD) patients. Given that heart disease is known to increase Alzheimer’s risk by ∼40%, we included the CV group because these patients will be at risk of AD by the vascular parameter. Clinical and demographic data were collected through questionnaires conducted during recruitments, while genetic testing for *ApoE* haplotypes was performed using the RT‐PCR‐based TaqMan SNP assay. Allelic and haplotype frequencies were determined.

**Result:**

In the CN group, the ε3ε4 haplotype was detected in 43 subjects (91.4%). Conversely, the ε3ε3 haplotype was found in three subjects (6.38%), while the least common haplotype, ε2ε4, was present in only one subject (2.12%), 95% CI [0.871, 1.04]. Within the CV cohort, 48 subjects were identified as carriers of the ε3ε4 haplotype (96%). Only one subject had the ε3ε3 haplotype (2%), and similarly, one subject harbored the ε2ε4 haplotype, 95% [0.943, 1.056]. In the AD group, the ε3ε4 haplotype was the most prevalent, with 44 carriers (91.6%). In this group, both the ε3ε3 and ε2ε4 haplotypes were each observed in one subject, collectively accounting for 4.16% of the cohort, 95% [0.915,1.08].

**Conclusion:**

Interestingly, our study revealed that the ε3 and ε4 alleles predominated across the various groups, while the ε2 allele emerged as the least common. Despite multiple reports linking the ε4 allele to an increased risk of AD, in our admixed population it seems that African ancestry enrichment at the *ApoE* locus potentially mitigates its effect. Further studies are warranted to elucidate the relationship between ancestry and AD risk.

